# Postprandial glycemic response in different ethnic groups in East London and its association with vitamin D status: Study protocol for an acute randomized crossover trial

**DOI:** 10.1177/02601060251356528

**Published:** 2025-07-08

**Authors:** Honglin Dong, Christian Reynolds, Saiful Islam, Swrajit Sarkar, Sophie Turner

**Affiliations:** 1 The School of Health and Medical Sciences, 698129 City St George's, University of London, London, UK; 2 Centre for Food Policy, 698129 City St George's, University of London, London, UK

**Keywords:** Postprandial glycemia, type 2 diabetes, oral glucose tolerance test, 25(OH)D, ethnic minority, body fat percentage, body mass index

## Abstract

**Background:**

In the UK, Black African-Caribbeans (ACs) and South Asians (SAs) have 3–6 times greater risks of developing type-2 diabetes mellitus (T2DM) and significantly higher prevalence of vitamin D (vitD) deficiency than White Caucasians. East London is among the areas with the highest prevalence of T2DM and the highest proportion of ethnic minority groups. This ethnic health inequality is ascribed to socioeconomic standing, dietary habits, culture, and attitudes, while biological diversity has rarely been investigated.

**Aim:**

The study aims to investigate the difference in the postprandial glycemic response (PGR), an independent risk factor of T2DM, between ethnic groups (White Caucasians, SAs, and ACs) in East London and its association with vitD status.

**Methods:**

This acute randomized crossover trial will recruit healthy adults (n = 106) in East London between November 2023 and March 2025. Two test drinks are consumed by participants (a glucose drink containing 75 g glucose and pure orange juice) on different occasions. PGRs are monitored before and after drinking every 30 min for up to 2 h via finger prick. A fasting blood sample obtained via phlebotomy will be used for plasma 25(OH)D and relevant tests. A knowledge/perception questionnaire about vitD and a 4-day food diary (analyzing vitD dietary intake) will also be collected. Data will be analyzed using a multiple linear regression model adjusted by confounding factors (age, gender, body mass index, and body fat percentage).

**Summary:**

The study results will be disseminated through journals and conferences, and target stakeholders.

## Introduction

Health patterns differ significantly between ethnic minority groups and the White population. In the UK, the risk of developing type 2 diabetes mellitus (T2DM) is 3–6 times greater in South Asians (SAs) and up to three times greater in Black African-Caribbeans (ACs) than in White Caucasians, and people in these groups develop this condition at a younger age (Meeks et al., 2016). East London is among the areas with the highest proportion of ethnic minority groups (Gov.uk, 2022a) and the highest prevalence of T2DM (Diabetes, 2021). Although multiple factors, including socioeconomic standing, diet, culture and attitudes, language barriers, genetics, and lifestyles, have been identified (Hanif and Susarla, 2018), research into biological diversity is scarce. Recent research revealed that the postprandial glucose peak in SAs is 2–3 times greater than in White Caucasians after identical carbohydrate loads are reached (Tan et al., 2017). Although obesity is believed to account for 90% of the risk of developing T2DM due to obesity causing insulin resistance (Grant et al., 2021) and some ethnic minority groups, e.g. Black people, have a higher prevalence of overweight and obesity than White British people do (73.6% vs. 63.3%) (Gov.uk, 2020), other biological mechanisms, including vitamin D (vitD) deficiency, are poorly understood.

VitD deficiency in ethnic minorities in the UK is well known and is described as an unrecognized epidemic (Darling, 2020). In the UK, 50% of SAs and 33% of Black ACs demonstrate vitD deficiency, whereas 17.5% of White Caucasians do (Sutherland et al., 2021), which is primarily due to more subcutaneous pigmentation that absorbs ultraviolet B from sunlight and reduces vitD production in the skin and at high latitudes in the UK (Yousef et al., 2023). This situation is worse in East London. In Tower Hamlets, a borough of East London, 80–97% of the residents are thought to have vitD deficiency, significantly higher than the national population at 50% (Towerhamlets.gov.uk, 2017). An inverse association of serum 25(OH)D levels with insulin resistance was observed in healthy adults (Yin et al., 2022) and diabetic patients (Abubaker et al., 2022). Recent evidence from systematic reviews and meta-analyses of randomized controlled trials (RCTs) suggests that vitD supplementation may help reduce fasting plasma glucose concentrations, HbA1c levels, and insulin resistance in patients with T2DM (Afraie et al., 2024; Farahmand et al., 2023). However, little evidence is available that focuses on ethnic minority groups or residents in East London, indicating that AC and SA communities are underrepresented in the evidence base concerning diabetes and vitD.

VitD plays an important role in calcium metabolism and is involved in the modulation of cell growth, neuromuscular and immune function, and the reduction of inflammation due to its receptors being expressed ubiquitously in nearly all human cells, including pancreatic β-cells (Lips et al., 2017). Animal studies have shown that vitD treatment improves insulin production and sensitivity via decreasing inflammatory response and regulating glucose homeostasis (Zhang et al., 2024), or via increasing intracellular calcium in pancreatic ß-cells and other metabolic pathways (Szymczak-Pajor et al., 2020). Moreover, 1,25(OH)_2_D (the active form of vitD) may modulate β-cell growth and differentiation (Lips et al., 2017). The secondary high parathyroid hormone (PTH) concentration (Malik et al., 2020) and increased inflammatory markers (Zhou and Hyppönen, 2023) associated with vitD deficiency may also cause glucose intolerance. VitD may have an indirect effect on glycemic control via obesity. Previous evidence (Rafiq and Jeppesen, 2018) revealed a significant inverse association between body mass index (BMI) and serum 25(OH)D, which is thought to involve a complex of mutual influences because vitD receptors are expressed on adipose cells and regulate their functions (Bennour et al., 2022), indicating that vitD deficiency might be one of the causes of obesity, thus indirectly leading to an increased risk of T2DM.

The postprandial glycemic response (PGR) has important implications for the development of T2DM (Shibib et al., 2024). The oral glucose tolerance test (OGTT) is widely used to assess insulin sensitivity and pancreatic β-cell function and to assess an individual's metabolic capacity to handle carbohydrate-containing foods (American Diabetes Association, 2013). However, a recently published study indicated that within-subject variations in the PGR pattern may exist between OGTT and food intake, suggesting the necessity of combining OGTT and a meal/drink tolerance test for individualized glycemic management (Liu et al., 2022). There is poor awareness about vitD and its impact on health in the UK. Although the COVID-19 pandemic has brought increased public attention to vitD, only around one in six individuals in the UK have been reported to take a daily vitD supplement (Gov.uk, 2022b). However, no such data is currently available for ethnic minority groups or residents of East London. We are also interested in dietary vitD intake between different ethnic groups in East London, which will partly explain the vitD status of the target population.

Overall, the evidence on the association of vitD and the risk factors of T2DM in the ethnic minority groups is scant. There is an urgent call for research on ethnic minority populations to address health inequality (The NHS Race and Health Observatory, 2021). This proposal aims to respond to the above call by focusing on ethnic minority communities in East London via investigating the association between vitD status and PGRs among White Caucasian, SA, and AC adults. The objectives of the study include (1) investigating PGRs to an OGTT, as well as to the consumption of a commonly consumed drink, pure orange juice (OJ); (2) measuring parameters in the blood including 25(OH)D (an indicator of vitD status), and other relevant tests including calcium, parathyroid hormone (PTH), blood lipid profile, inflammatory parameters; (3) assessing the knowledge and perception of vitD; (4) evaluating the dietary vitD intake, in White Caucasian, SA and AC adults.

## Method

**Figure 1.**
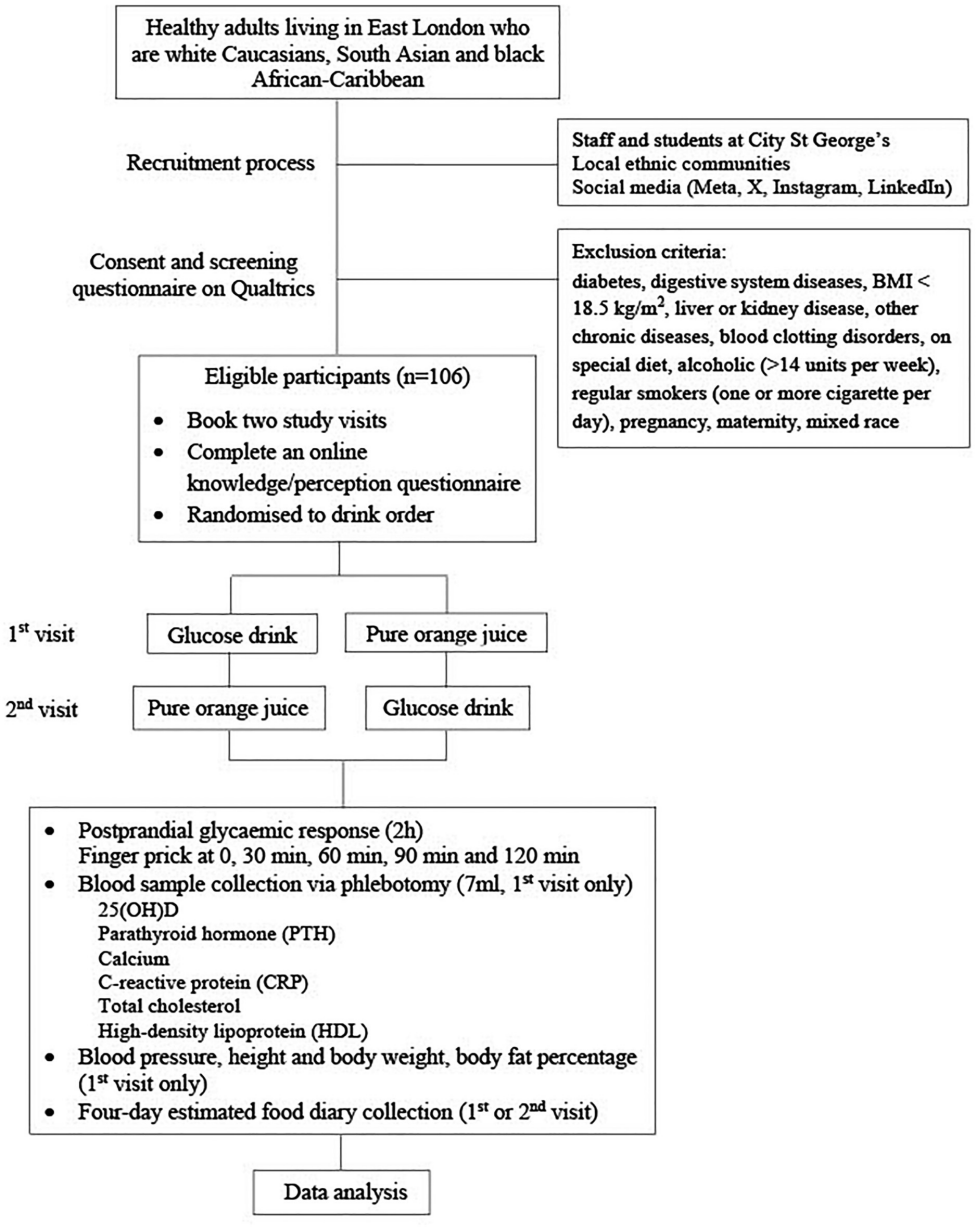
Study flow diagram.

This is an acute, randomized, repeated measures crossover trial. Figure 1 shows the study flow chart. The proposal was approved by the Senate Research Ethics Committee at City St George's, University of London (Ref ETH2223-2000). This study was registered with ClinicalTrials.gov (identifier: NCT06241976). The recruitment period of the study is between 1 November 2023 and 31 March 2025 in London, UK. The informed consent is obtained from all participants before taking part in the study.

Study status: A) participant recruitment will be completed at the end of March 2025; B) data collection will be completed at the end of March 2025; C) results are expected in April and May 2025. None of these stages have already been completed when the manuscript was submitted.

## Participants

The inclusion criteria are as follows: 18–65 years in general good health and living in East London from White, SA or Black AC origins. The exclusion criteria are as follows: diabetes; digestive system diseases; BMI < 18.5 kg/m^2^; liver or kidney disease; other chronic diseases; blood clotting disorders; alcohol consumption (>14 units per week); regular smoking (one or more cigarettes per day); pregnancy; maternity; and mixed race. A self-developed health and lifestyle questionnaire (Appendix 1) will be used to screen the eligibility of the participants. Participants will provide informed consent online before being screened for eligibility.

## Recruitment

There are a few methods of recruiting participants. We will recruit staff and students who live in East London with gatekeeper permissions from the Dean of the school. Recruitment adverts will be circulated to staff and students at City St George's, University of London. There are 19975 students, among whom 64% are from the UK, and a large proportion of students are from different London boroughs, including the East London area. Each year, many staff and student projects recruit participants successfully in this way. In addition, we recruit participants from local communities in East London with gatekeeper permission. We will contact local ethnic communities, including the Bangladeshi Community, London Central Mosque, Bangladesh Embassy and Indian & Bangladesh Hindu Community East London, etc. We will also recruit participants via social media, including Meta (formerly Facebook), X (formerly Twitter), Instagram, and LinkedIn. To encourage taking part in the study, participants will receive a shopping voucher worth £20 per visit as a gesture of thanks and an incentive.

## Study design

The participants will consume a glucose drink (75 g glucose in 300 ml water, 281 kcal) used for the OGTT (Eyth et al., 2023) and pure OJ (Tesco 100% Pure Squeezed Orange Juice Smooth 300 ml containing 129 kcal, 30 g sugar, 0.3 g fiber, 1.8 g protein and 90 mg vitamin C) on separate occasions with at least 48-h interval and at random order. The two drinks were chosen rather than meals because of fewer facilities needed to cater to participants, fewer potential food hygiene issues, and being more acceptable to participants from different ethnic backgrounds. The participants fast for 8–12 h. The blood glucose concentration is measured via a HemoCue Glucose 201+ Analyzer (Health-care Equipment & Supplies, Surrey, UK) at 0, 30, 60, 90, and 120 min before and after drink consumption by finger prick. Seven milliliters of fasting blood will be collected via phlebotomy on the first visit. If blood collection is unsuccessful on the first visit, it will be attempted on the second visit. The plasma is separated by centrifuging the blood sample at 2000 × g for 10 min and stored at −20°C until analysis of the metabolic parameters (refer to the section on outcome measures).

Participants are asked to consume the drinks (either a glucose drink or OJ) within 5 min in the resting area outside the clinic room. The researcher will record the start and end times of consumption. During the 2-h study period, the participants are asked to remain sedentary and refrain from eating or drinking while sitting in the resting area. On the evening before each study visit, participants are encouraged to consume similar meals across both visits, ensure adequate sleep, and avoid alcohol consumption and strenuous physical activity. On the study visit, the researcher will collect information on the participant's dinner from the previous evening, hours of sleep, alcohol intake, and physical activity.

The name, email/mobile (for appointment purposes), sex, age, and ethnicity of the participants are also collected. The research assistant (ST) will generate the allocation sequence, enroll participants, collect consent forms, assign participants to interventions and conduct the trial. The study is not blind to the researcher and participants but is blind to the statistician (SI) who will analyze the data.

## Randomization

The order of drink consumption is randomized by using the Excel RAND function. In an Excel spreadsheet, a list of participant numbers is shown in one column. In the next column, we use the RANDBETWEEN function and choose 0 and 1 as the ranges to randomly generate values of 0 or 1. Participants with a value of 0 will consume a glucose drink first, while participants with a value of 1 will consume pure OJ as their first drink.

## Outcome measures

The primary outcome will be the association of plasma 25(OH)D with the PGRs represented as the incremental areas under the curve (iAUCs) and peak values (PVs) in different ethnic groups. The secondary outcomes include the difference in iAUCs, PVs, and the following variables relating to vitD status between ethnic groups. C-reactive protein (CRP) is reported to have an inverse relationship with 25(OH)D, indicating an anti-inflammatory property of vitD (de Oliveira et al., 2017). The total cholesterol and high-density lipoprotein (HDL) have inverse and positive associations, respectively, with vitD status (Alkhatatbeh et al., 2019). BMI and body fat percentage are used as confounding factors of 25(OH)D (Grønborg et al., 2015) and PGRs to drinks (Dhaheri et al., 2010). PTH and calcium are closely related to vitD metabolism (Goltzman et al., 2018). The plasma 25(OH)D and PTH are measured using an AIA-900 immunoassay analyzer (Tosoh Bioscience, USA); CRP, calcium, total cholesterol, and HDL are measured using Horiba Pentra 400 Biochemistry Analyzer (Horiba, Japan). The body weight and fat percentage are measured with dual-frequency bioelectrical impedance analysis technology by TANITA DC-360 P (Tanita, Amsterdam) and body height by stadiometer. Dietary vitD intake will be analyzed by a 4-day estimated food diary analyzed using Nutritics software (Nutritics Ltd, Dublin). Knowledge and perception of vitD will be assessed by an online questionnaire (Appendix 2) collected via Qualtrics survey platform (Qualtrics, Provo, UT).

## Data analysis

The sample size was calculated using G*Power software (version 3.1.9.7; Heinrich Heine University, Düsseldorf, Germany), based on F-tests for ANOVA: repeated measures, between-subjects factors. This study aims to achieve an effect size of 0.25 in the PGRs among three ethnic groups, considering that the response is taken from five different time points with 30-min intervals for each person, and to achieve 80% power in the study, we will need 32 people in each group (n = 96 in total) at the 5% level of significance. The details of the sample size calculation can be found in Appendix 3. Considering the acute nature of the study, we believe 10% of the attention is sufficient; therefore, the target number of recruitments is 106 participants. Data normality will be assessed using the Kolmogorov–Smirnov test. Continuous variables with a normal distribution will be presented as means ± standard deviations, while non-normally distributed data will be reported as medians and interquartile ranges. Categorical data are presented as numbers and percentages. The iAUC is calculated using approximated trapezoidal numerical integration (Chlup et al., 2008), and only the incremental area above the fasting level will be included. The association of plasma 25(OH)D concentrations and the iAUCs and PVs, respectively, will be analyzed using a multiple linear regression model adjusted by age, gender (using dummy variables), BMI, and body fat percentages, including all participants, as well as in different ethnic groups. The iAUCs and PVs concentrations between ethnic groups after each drink consumption will be analyzed by one-way ANCOVA adjusted by confounding factors including fasting glucose concentration, age, sex, BMI, and body fat percentage. The correlation of iAUCs and PVs between the two drink consumptions is analyzed by Pearson's correlation to identify the difference in the pattern of PGRs to different drink consumptions. Other continuous variables (e.g. plasma 25(OH)D etc.) between ethnic groups will be analyzed by one-way ANCOVA adjusting confounding factors (age, gender, BMI and body fat percentage). Categorical variables, e.g. gender, the percentage of patients with vitD deficiency etc., are compared between ethnic groups via Chi-square tests. The statistical significance will be reported with a *P*-value (significance was set at *P* ≤ 0.05, two-tailed) and a 95% confidence interval. The statistical software IBM SPSS 29 will be used to analyze the data. The percentage of missing data is expected to be low based on our previous experience. Therefore, regression imputation is used to address missing data. Both intention-to-treat analysis and per-protocol analysis will be used for the data analysis.

## Advisory board

An advisory board will include senior staff members from the Ethics Committee and the research centers at the school and the principal investigator. The advisory board will meet every 3 months and make sure the following are checked and in place: safeguard participants in research; protect researchers/investigators (by providing a clear framework to work within); enhance ethical and scientific quality; minimize risk; monitor practice and performance; promote good practice and ensure lessons are learned.

## Dissemination of the study

The findings of the study will be communicated to other researchers, clinical professionals, and policymakers primarily through publications in peer-reviewed journals, seminars, and conferences. A summary leaflet will be produced in plain English and shared with participants, local communities, GP clinics, and hospitals. The leaflet will include actions that can be taken by East London residents in their local food environments. The findings will also be disseminated through university newsletters and the university's official social media accounts, including Meta, X, Instagram, LinkedIn, and YouTube.

## Implications of the study

The study outcomes will help address the existing research gap regarding the relationship between vitD, PGRs and ethnicities in the UK. The study will raise awareness of the health implications of vitD deficiency, particularly its association with T2DM, and provide rationales to inform educational programs and food fortification strategies aimed at addressing vitD deficiency in ethnic minority populations in East London and beyond. Though the study is observational in nature, it is anticipated that a major outcome of this study will be evidence to inform a RCT to confirm the causal relationship of vitD status and glycemic control in ethnic minorities in the UK. Currently, research on vitD supplementation and PGR interventions has produced inconclusive results, and research on ethnic minority groups is needed (Christides, 2022).

## Limitations and future perspectives

**Figure 2.**
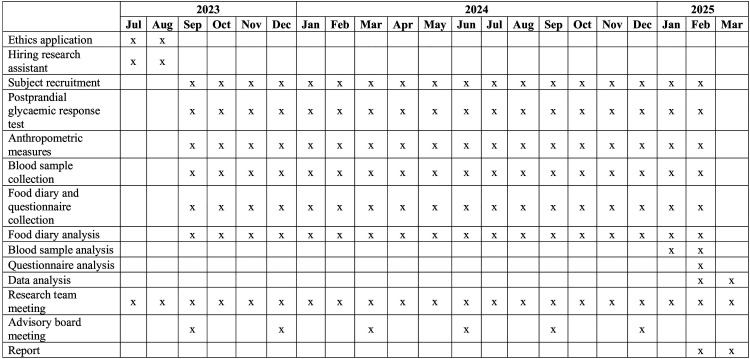
Gantt chart.

There are several limitations to this proposal. Firstly, the use of multiple finger-prick tests to measure postprandial blood glucose concentrations at 30-min intervals may miss the actual peak glucose value. Using 15-min sampling interval or continuous glucose monitoring (CGM) (Shields et al., 2024) would obtain more precise PGRs. Secondly, the proposal focuses on participants residing in East London; therefore, findings related to knowledge and perceptions of vitD, dietary intake, and the prevalence of deficiency may have limited generalizability to populations outside of this geographic area. In future studies, CGM (Shields et al., 2024) could replace finger-prick testing to assess PGRs following meal or drink consumption. This approach would provide more accurate and comprehensive data while reducing participant burden, provided budget constraints are not a limitation. Additionally, a large-scale study involving a more representative sample of diverse ethnic groups recruited from multiple London boroughs is warranted to enhance the generalizability of the findings. Data management can be found in Appendix 4 and timelines (Gantt Chart) can be found in Figure 2.

## Supplemental Material

sj-docx-1-nah-10.1177_02601060251356528 - Supplemental material for Postprandial glycemic response in different ethnic groups in East London and its association with vitamin D status: Study protocol for an acute randomized crossover trial Supplemental material, sj-docx-1-nah-10.1177_02601060251356528 for Postprandial glycemic response in different ethnic groups in East London and its association with vitamin D status: Study protocol for an acute randomized crossover trial by Honglin Dong, Christian Reynolds, Saiful Islam, Swrajit Sarkar and Sophie Turner in Nutrition and Health

sj-docx-2-nah-10.1177_02601060251356528 - Supplemental material for Postprandial glycemic response in different ethnic groups in East London and its association with vitamin D status: Study protocol for an acute randomized crossover trial Supplemental material, sj-docx-2-nah-10.1177_02601060251356528 for Postprandial glycemic response in different ethnic groups in East London and its association with vitamin D status: Study protocol for an acute randomized crossover trial by Honglin Dong, Christian Reynolds, Saiful Islam, Swrajit Sarkar and Sophie Turner in Nutrition and Health

sj-docx-3-nah-10.1177_02601060251356528 - Supplemental material for Postprandial glycemic response in different ethnic groups in East London and its association with vitamin D status: Study protocol for an acute randomized crossover trial Supplemental material, sj-docx-3-nah-10.1177_02601060251356528 for Postprandial glycemic response in different ethnic groups in East London and its association with vitamin D status: Study protocol for an acute randomized crossover trial by Honglin Dong, Christian Reynolds, Saiful Islam, Swrajit Sarkar and Sophie Turner in Nutrition and Health

sj-docx-4-nah-10.1177_02601060251356528 - Supplemental material for Postprandial glycemic response in different ethnic groups in East London and its association with vitamin D status: Study protocol for an acute randomized crossover trial Supplemental material, sj-docx-4-nah-10.1177_02601060251356528 for Postprandial glycemic response in different ethnic groups in East London and its association with vitamin D status: Study protocol for an acute randomized crossover trial by Honglin Dong, Christian Reynolds, Saiful Islam, Swrajit Sarkar and Sophie Turner in Nutrition and Health

sj-docx-5-nah-10.1177_02601060251356528 - Supplemental material for Postprandial glycemic response in different ethnic groups in East London and its association with vitamin D status: Study protocol for an acute randomized crossover trial Supplemental material, sj-docx-5-nah-10.1177_02601060251356528 for Postprandial glycemic response in different ethnic groups in East London and its association with vitamin D status: Study protocol for an acute randomized crossover trial by Honglin Dong, Christian Reynolds, Saiful Islam, Swrajit Sarkar and Sophie Turner in Nutrition and Health

sj-doc-6-nah-10.1177_02601060251356528 - Supplemental material for Postprandial glycemic response in different ethnic groups in East London and its association with vitamin D status: Study protocol for an acute randomized crossover trial Supplemental material, sj-doc-6-nah-10.1177_02601060251356528 for Postprandial glycemic response in different ethnic groups in East London and its association with vitamin D status: Study protocol for an acute randomized crossover trial by Honglin Dong, Christian Reynolds, Saiful Islam, Swrajit Sarkar and Sophie Turner in Nutrition and Health
