# Supplementary material for: Postprandial glycemic response in different ethnic groups in East London and its association with vitamin D status: Study protocol for an acute randomized crossover trial
Source: Nutr Health. 2025 Jul 8;31(4):1307–13. doi: 10.1177/02601060251356528 (PMC12660509; doi:10.1177/02601060251356528)
Supplement: sj-docx-1-nah-10.1177_02601060251356528 - Supplemental material for Postprandial glycemic response in different ethnic groups in East London and its association with vitamin D status: Study protocol for an acute randomized crossover trial [file sj-docx-1-nah-10.1177_02601060251356528.docx]

**Appendix 1 Health and lifestyle questionnaire**

**Health and Lifestyle Questionnaire (screening)**

| **Name:** | |
| --- | --- |
| **Age:** | **Gender:** |
| **Ethnicity:** | **Email:**  **Phone:** |

**Signature of subject Date:**

**Thank you for completing this questionnaire**
