# Supplementary material for: Postprandial glycemic response in different ethnic groups in East London and its association with vitamin D status: Study protocol for an acute randomized crossover trial
Source: Nutr Health. 2025 Jul 8;31(4):1307–13. doi: 10.1177/02601060251356528 (PMC12660509; doi:10.1177/02601060251356528)
Supplement: sj-docx-2-nah-10.1177_02601060251356528 - Supplemental material for Postprandial glycemic response in different ethnic groups in East London and its association with vitamin D status: Study protocol for an acute randomized crossover trial [file sj-docx-2-nah-10.1177_02601060251356528.docx]

**Appendix 3 Sample size calculation using G*Power**

**F tests -** ANOVA: Repeated measures, between factors

**Analysis:** A priori: Compute required sample size

**Input:** Effect size f = 0.25

α err prob = 0.05

Power (1-β err prob) = 0.80

Number of groups = 3

Number of measurements = 5

Corr among rep measures = 0.5

**Output:** Noncentrality parameter λ = 10.0000000

Critical F = 3.0943374

Numerator df = 2.0000000

Denominator df = 93.0000000

Total sample size = 96

Actual power = 0.8020225
