# Supplementary material for: Postprandial glycemic response in different ethnic groups in East London and its association with vitamin D status: Study protocol for an acute randomized crossover trial
Source: Nutr Health. 2025 Jul 8;31(4):1307–13. doi: 10.1177/02601060251356528 (PMC12660509; doi:10.1177/02601060251356528)
Supplement: sj-docx-3-nah-10.1177_02601060251356528 - Supplemental material for Postprandial glycemic response in different ethnic groups in East London and its association with vitamin D status: Study protocol for an acute randomized crossover trial [file sj-docx-3-nah-10.1177_02601060251356528.docx]

**Appendix 4 Data Management**

**Postprandial glycaemic response in different ethnic groups in East London and its association with vitamin D status**

# Data Collection

## What data will you collect or create?

The following data will be collected:

Demographic information: age, gender, ethnicity

Clinical biochemical measures: Serum 25(OH)D, PTH, calcium, fasting blood glucose (Glu), postprandial blood glucose, cholesterol, high density lipoprotein (HDL), C-reactive protein (CRP), Triglycerides (TG)

Anthropometric measures: height, weight, waist circumference, body fat

A questionnaire about the knowledge and perceptions of vitamin D

tabular data, survey data, experimental measurements, models, software etc.

For the purpose of data sharing, reuse and preservation into the future, the following types of the data will be used.

Text files - MS Word docs, PDF Numerical - SPSS, Stata, Excel

Software - Nutritics analytical software, RefWorks

The following formats of the data were chosen based on the standards accepted by data centres or widespread usage within a given community.

Comma-separated values (.csv). Tab-delimited file (.tab).

Delimited text with SQL data definition statements.

## How will the data be collected or created?

The data will be organised during the project, including naming conventions (e.g. participant ID plus study visit number followed by name of the parameters (or their abbreviations), version control (add editor's name initials and date) and folder structures. The consistency and quality of data collection will be controlled and documented via calibration, repeat samples or measurements, data entry validation, peer review of data.

# Documentation and Metadata

## What documentation and metadata will accompany the data?

This will include the names who created or contributed to the data, its title, date of creation and under what conditions it can be accessed, details on the methodology used, analytical and procedural information, definitions of variables, units of measurement, any assumptions made, and the format and file type of the data. For each measure, the above information will be populated in a separate document (a template will be produced and applied to all other measures). The document will be included in the folder of the relevant measure.

# Ethics and Legal Compliance

## How will you manage any ethical issues?

The ethics application is submitted to the Senate Research Ethics Committee at City St George’s, University of London. The following are ethics considerations.

**Informed consent:** Participants will be provided an information sheet to fully explain the procedure of the study using plain English language. All participants need to give written consent before taking part in the study.

**Voluntary participation**: Participants can withdraw from the study at any time without giving any reasons.

**Anonymity:** During the data collection phase, participants will not be anonymous because they will provide their name and contact email or phone number for the purpose of making appointments. Participants will be given a unique identifier code and their contact information will be stored separately from collected data. Once data have been collected, only the unique identifier will be used and data will be anonymised.

**Confidentiality:** All data will be stored in a locked cabinet (hard copy) or OneDrive with password protection. Only researchers can access the data collected from participants. If the study is published no individual identity will be revealed.

**Potential for harm:** The participants will give a blood sample via phlebotomy which may leave a bruise. There will be multiple blood sample collections via finger prick. We will exclude people who have blood clotting problems. In addition, there are some burdens for participants recording a 4-day food diary. There is no other harm to participants.

**Results communication:** The findings of the study will be published in peer-reviewed journals. The paper will be sent to participants upon request. This is indicated in the participant information sheet.

## How will you manage copyright and Intellectual Property Rights (IPR) issues?

Both City St George’s, University of London and the funder will own the copyright and IPR of any data that are collected or created. IPR ownership may be covered in a consortium agreement.

# Storage and Backup

## How will the data be stored and backed up during the research?

The data will be stored in MS Office 365 OneDrive with an account with City St George’s, University of London. It can store **1TB of data.**

Automatic back-up will be set up for all data by the PI.

## How will you manage access and security?

We will follow UK Data Service guidance on [data security](https://www.ukdataservice.ac.uk/manage-data/store/security) to manage data access and security.

# Selection and Preservation

## Which data are of long-term value and should be retained, shared, and/or preserved?

All raw data and processed data will be preserved for long-term re-use. Personal data of participants and consent forms will be deleted safely in 5 years after the project finishes.

## What is the long-term preservation plan for the dataset?

The datasets will be deposited at the City’s data depository Figshare <https://city.figshare.com/account/home>

# Data Sharing

# How will you share the data?

Figshare is used to store and share confidential data as well as publishing open-access data for future use.

## Are any restrictions on data sharing required?

No

# Responsibilities and Resources

## Who will be responsible for data management?

The principal investigator will be responsible for data management

## What resources will you require to deliver your plan?

Data management training
