# Supplementary material for: Postprandial glycemic response in different ethnic groups in East London and its association with vitamin D status: Study protocol for an acute randomized crossover trial
Source: Nutr Health. 2025 Jul 8;31(4):1307–13. doi: 10.1177/02601060251356528 (PMC12660509; doi:10.1177/02601060251356528)
Supplement: sj-docx-4-nah-10.1177_02601060251356528 - Supplemental material for Postprandial glycemic response in different ethnic groups in East London and its association with vitamin D status: Study protocol for an acute randomized crossover trial [file sj-docx-4-nah-10.1177_02601060251356528.docx]

Name of principal investigator/researcher: Dr Honglin Dong

# REC reference number: ETH2223-2000

# Title of study: Postprandial glycaemic response in different ethnic groups in East London and its association with vitamin D status

Please tick

or initial box

| 1 | I confirm that I have read and understood the participant information dated [13^th^ April 2023] for the above study. I have had the opportunity to consider the information and ask questions which have been answered satisfactorily. |  |
| --- | --- | --- |
| 2 | I understand that my participation is voluntary and that I am free to withdraw without giving a reason without being penalised or disadvantaged. |  |
| 3 | I understand that I will be able to withdraw my data until the data are de-identified (4 weeks after your participation) or until I submit my anonymous questionnaire online or hard copy. |  |
| 4 | I agree to provide some of my personal information including name, contact number and email address for the purpose of making appointments. |  |
| 5 | I agree to City St George’s, University of London recording and processing this information about me. I understand that this information will be used only for the purpose(s) explained in the participant information and my consent is conditional on City St George’s, University of London complying with its duties and obligations under the General Data Protection Regulation (GDPR). |  |
| 5 | I would like to be informed of the results of this study once it has been completed and understand that my contact details will be retained for this purpose. |  |
| 6 | I confirm that I understand that my anonymous data may share with the funder of the project (Barts Charity) and make published at open access journals to meet funding requirements. |  |
| 7 | I agree to take part in the above study. |  |

____________________ ____________________________ _____________

Name of Participant Signature Date

____________________ ____________________________ _____________

Name of Researcher Signature Date

When completed, 1 copy for participant; 1 copy for researcher file.

Thank you for participating in the study

**PARTICIPANT INFORMATION**

# **REC reference number: ETH2223-2000**

# **Date and version of information sheet: 13/04/2023**

# **Title of study**

Postprandial glycaemic response in different ethnic groups in East London and its association with vitamin D status

# **Name of principal investigator/researcher:** Dr Honglin Dong

# **Invitation paragraph**

We would like to invite you to take part in a research study investigating whether there is an association of vitamin D deficiency with one of the risk factors of developing type-2 diabetes, postprandial glycaemic response, which means your blood glucose concentration change after you consume food or drink. We will recruit healthy adults who are white Caucasians, South Asians and black African -Caribbeans and are residents in East London.

Before you decide whether you would like to take part it is important that you understand why the research is being done and what it would involve for you. Please take time to read the following information carefully and discuss it with your family members or friends if you wish. Ask us if there is anything that is not clear or if you would like more information. You will be given a copy of this information sheet to keep.

# **What is the purpose of the study?**

Vitamin D deficiency is known to be more prevalent in people with darker skin including African -Caribbeans and South Asians. East London is among the top areas in London that has the highest proportion of minority groups. Meanwhile, East London has some of the highest prevalence of diabetes in the country including Redbridge (8.66%) and Newham (8.57%). Evidence shows that vitamin D plays important roles in insulin sensitivity so as to prevent people from developing insulin resistance thus type-2 diabetes. However, little evidence is available for minority groups or residents in East London, indicating East London communities being underrepresented in the evidence base around diabetes and vitamin D.

The aim of the study is to investigate whether there is a relationship between vitamin D deficiency and one of the risk factors of developing type-2 diabetes, postprandial glycaemic response in the ethnic groups including white Caucasians, South Asians and African-Caribbeans.

# **Why have I been invited to take part?**

We are recruiting 126 healthy adults (18-65 years old) who are residents in any boroughs of East London, and who are from white, African -Caribbean or South Asian origins. However you are not eligible to take part in the study if you have diabetes, digestive system diseases, body mass index (BMI) < 18.5 kg/m2 (you can calculate your BMI by the formular of body weight (kg) divided by the square of your height in meter), liver or kidney disease, other chronic diseases, blood clotting disorders, consume alcohol more than 14 units per week, regular smokers (one or more cigarette per day), fasting blood glucose ≥ 11 mmol/L (we will test this on your first visit). If you are students, either taking part or not taking part in the study will have no effect on assessments, marks or future studies. If you are employees (not just City St George’s, University of London), choosing to either take part or not take part will have no effect on your employment or promotion prospects etc.

# **Do I have to take part?**

Participation in the study is voluntary, and you can choose not to participate in part or all of the project. You can withdraw at any stage of the project without being penalised or disadvantaged in any way. It is up to you to decide whether or not to take part. If you do decide to take part, you will be asked to sign a consent form. If you decide to take part, you are still free to withdraw at any time and without giving a reason.

If you take part in the study, you can withdraw your data until the data has been de-identified. All your data collected up to the point of withdrawal will be deleted from the dataset.

**What will happen if I take part?**

You will be asked to fill in a health and lifestyle screening questionnaire that will be sent to you via email. A hard copy will be available to collect at the Northampton Square at City St George’s, University of London (venue will be confirmed with you in due course) if you wish. If you are eligible for the study, you will need to sign the consent form and send it to the researcher via email ([honglin.dong@citystgeorges.ac.uk](mailto:honglin.dong@city.ac.uk)) or in-person (you can do this on your first visit), or post it to the researcher (Dr Honglin Dong, Myddelton Building, 1 Myddelton St, London EC1R 1UB). You will need to come to a research lab at Northampton Square campus at City St George’s, University of London twice, with at least 48 hours of interval. The researcher will discuss with you about the time and date for the two visits via either email or mobile phone.

For each visit, you are asked to be fasting for at least 8 hours prior to your appointment (normally in the morning between 8-10am). You will consume one of the two drinks at separate occasions (visits), one is glucose drink (75g glucose in 300ml water), another is pure orange juice 300ml. You are asked to complete each drink consumption within 5 min and asked to keep the time consistent between the two drinks (the time used to consume the drink will be recorded). Your blood glucose concentration is measured at 0 (fasting), 30, 60, 90 and 120 min by finger prick before and after drink consumption. During the 2-h study period, you are asked to keep sedentary (recommended to bring a book or your laptop to kill the time), not eat and drink anything. At the night prior to your study visit, you should follow your normal diet, have a good night's sleep and avoid alcohol and intensive exercises.

On your first visit only, apart from the above activities, a 7 ml fasting blood (equivalent to around 1.5 teaspoons) is collected via phlebotomy (which means that a needle is used to take blood from a vein in your arm) by trained professionals to measure your plasma 25(OH)D (a metabolite of vitamin D that is an indicator of your vitamin D status), and some other biochemical tests relating to vitamin D status including C-reactive protein (CRP), parathyroid hormone (PHT), calcium, cholesterol and high-density lipoprotein (HDL). Your height and weight, and your body fat composition will be also measured. In addition, you are asked to fill in an anonymous questionnaire on your knowledge and perception about vitamin D (around 10 min) and provide a 4-day food diary (four consecutive days including a weekend day). The food diary example and template will be provided to you either via email or hard copy. If you choose using hard copy, please return the photocopied version to the researcher via email or in-person. Your name, mobile number, email address will be collected for the purpose of making appointments and inform you of the study results once the study is complete. Your age, gender and ethnicity will be also collected for the study use only.

As a gesture of thanks, you will receive a total of £40 worth of voucher (£20 per visit). The voucher will be given on your last visit). However, you will have to pay the travel cost for the two visits.

Please be aware that you will be able to withdraw my data until the data are de-identified (4 weeks after your participation) or until you submit your anonymous questionnaire online or hard copy. Please also be minded that the study is for research rather than diagnostic purpose. However, we will advise you of contacting your GP if there are any concerns in your blood glucose levels.

# **What are the possible disadvantages and risks of taking part?**

# There is no possible disadvantage or risk of taking part in the study. However, we will take multiple finger pricks (5 times in total for each of the study visits, 2 visits in total with at least 48 hours of interval). We will also take a blood sample of 7 ml via phlebotomy only on the first visit). There might be left bruise after phlebotomy. The researcher who will do the blood collection is trained professionally.

# **What are the possible benefits of taking part?**

# There is no direct benefit of taking part in the study. However, your participation will be an important contribution to the knowledge of the relationship of vitamin D and type-2 diabetes risks. By joining the study, you and your family/friends as well as the wider communities in East London will raise the awareness of vitamin D deficiency and learn how to increase your vitamin D intake via diet or exposure to sunlight.

# **How is the project being funded?**

# The study is funded by Barts Charity (Award reference G-002602).

# **Conflicts of interests**

# There are no any conflicts held by researcher / research team / organisation / funder.

**What should I do if I want to take part?**

If you are interested in the study, please contact the researcher (Dr Honglin Dong, [honglin.dong@citystgeorges.ac.uk](mailto:honglin.dong@city.ac.uk)). Dr Dong will send you the participant information sheet with details of the study, a health and lifestyle questionnaire to evaluate your eligibility of taking part in the study and a consent form will be sent to you via email.

# **Data privacy statement**

# City St George's, University of London is the sponsor and the data controller of this study based in the United Kingdom. This means that we are responsible for looking after your information and using it properly. The legal basis under which your data will be processed is City St George's, University of London’s public task.

Your right to access, change or move your information is limited, as we need to manage your information in a specific way in order for the research to be reliable and accurate. To safeguard your rights, we will use the minimum personal-identifiable information possible (for further information please see [Information Commissioner's Office-Public task](https://ico.org.uk/for-organisations/guide-to-data-protection/guide-to-the-general-data-protection-regulation-gdpr/lawful-basis-for-processing/public-task/?q=privacy+notice)).

City St George's University of London will use your name and contact details to contact you about the research study as necessary. If you wish to receive the results of the study, your contact details will also be kept for this purpose. The only people at City St George’s, University of London who will have access to your identifiable information will be the researchers in this study. City St George's, University of London will keep identifiable information about you from this study for 3 years after the study has finished.

You can find out more about how City St George's, University of London handles data by visiting [Data Protection Policy](https://www.city.ac.uk/about/governance/policies/data-protection-policy) and [General Privacy Notice](https://www.city.ac.uk/about/governance/policies/general-privacy-notice). If you are concerned about how we have processed your personal data, you can contact the Information Commissioner’s Office (IOC) <https://ico.org.uk/>.

# **Will my taking part in the study be kept confidential?**

Only the researchers in the study have access to your personal data. All the personal identifiable information will be de-identified in the dataset by allocating each participant an ID number. All digital data collected from participants will be stored at University OneDrive. Hard copies of data will be stored in a locked filing cabinet that only researchers have access to. The de-identified data will be published in the UK Data Archive or Figshare in line with Open Scholarship principles.

We may use your contact details to inform you of a new study that you may wish to take part in.

# **What will happen to the results?**

# We will publish the findings of the study in journals and/or present the results in a national or international conference once the study has finished, in which cases anonymity will be maintained. If you wish to receive a copy of the publication/summary of the results, we will use your contact details to reach you and send them to you via email.

# **Who has reviewed the study?**

# This study has been approved by the Senate Research Ethics Committee at City St George’s, University of London.

# **What if there is a problem?**

# If you have any problems, concerns, or questions about this study, you should ask to speak to a member of the research team. If you remain unhappy and wish to complain formally, you can do this through City St George’s, University of London’s complaints procedure. To complain about the study, you need to phone 020 7040 3040. You can then ask to speak to the Secretary of the Senate Research Ethics Committee and inform them that the name of the project is [Postprandial glycaemic response in different ethnic groups in East London and its association with vitamin D status].

You can also write to the Secretary at:

Annah Whyton

Research & Enterprise Office

City St George’s, University of London

Northampton Square, London, EC1V 0HB

Email: [senaterec@citystgeorges.ac.uk](mailto:senaterec@city.ac.uk)

# **Insurance**

# City St George’s, University London holds insurance policies which apply to this study, subject to the terms and conditions of the policy. If you feel you have been harmed or injured by taking part in this study, you may be eligible to claim compensation. This does not affect your legal rights to seek compensation. If you are harmed due to someone’s negligence, then you may have grounds for legal action.

# **Further information and contact details**

# If you have any inquiries about the research, please contact:

Honglin Dong, via email [honglin.dong@citystgeorges.ac.uk](mailto:honglin.dong@city.ac.uk) or phone 020 7040 4053.

Sophie Turner, via email [sophie.turner.3@citystgeorges.ac.uk](mailto:sophie.turner.3@city.ac.uk) or mobile phone 07867255366.

**Thank you for taking the time to read this information sheet.**
