# Supplementary material for: Postprandial glycemic response in different ethnic groups in East London and its association with vitamin D status: Study protocol for an acute randomized crossover trial
Source: Nutr Health. 2025 Jul 8;31(4):1307–13. doi: 10.1177/02601060251356528 (PMC12660509; doi:10.1177/02601060251356528)
Supplement: sj-docx-5-nah-10.1177_02601060251356528 - Supplemental material for Postprandial glycemic response in different ethnic groups in East London and its association with vitamin D status: Study protocol for an acute randomized crossover trial [file sj-docx-5-nah-10.1177_02601060251356528.docx]

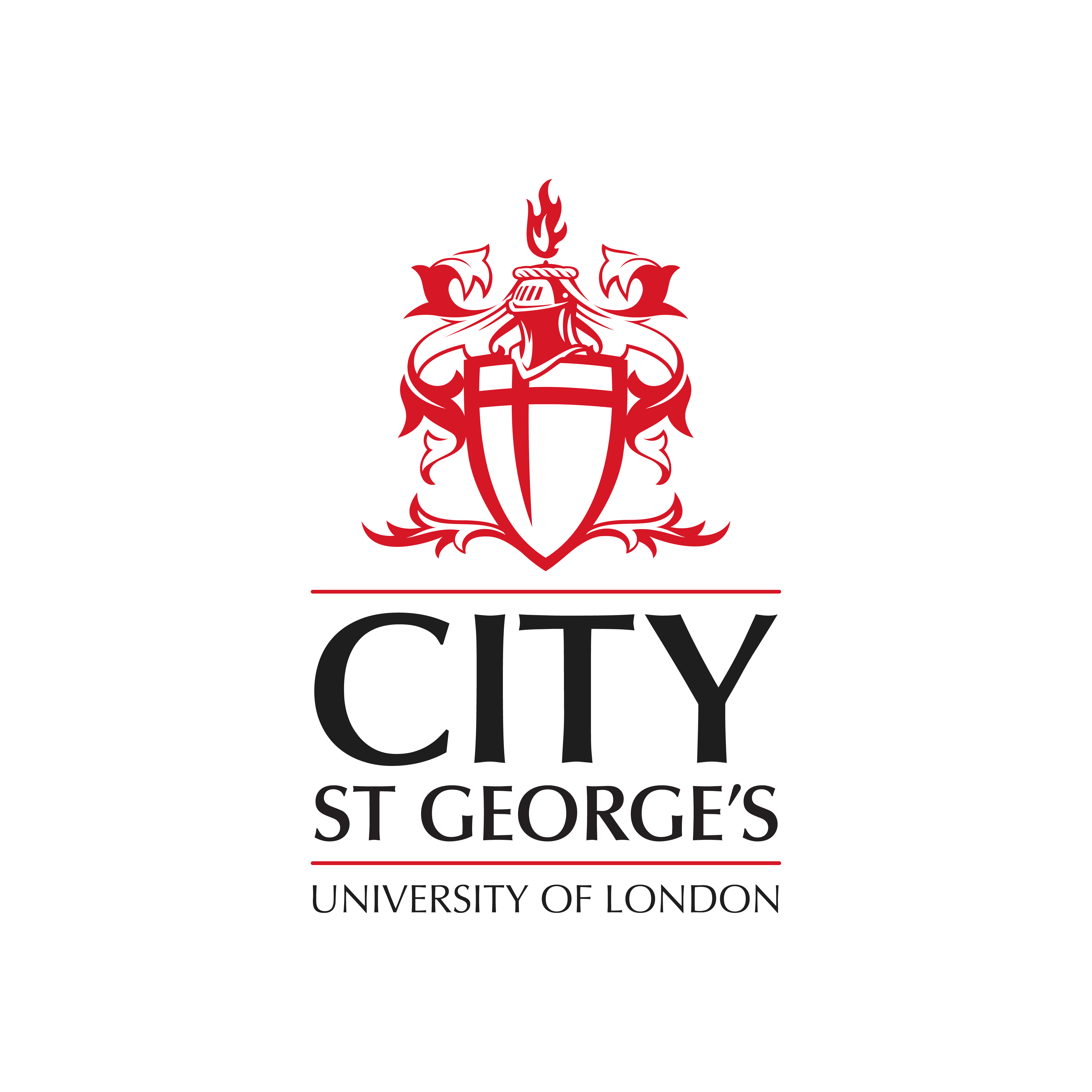


**Health & Safety Risk Assessment Form**

| **School/Professional Service** | | School of Health and Medical Sciences | | | | **Highest Risk Rating**  (given existing controls) | | **High** |  |
| --- | --- | --- | --- | --- | --- | --- | --- | --- | --- |
| **Room/Equipment/Activity Location(s)** | | DLG30D, Rhind Building | | | |  |  | **Medium** |  |
| **Head of Department/Principal Investigator Responsible for the Area/Task** | | Angeliki Bogosian/Honglin Dong | | | |  |  | **Low** |  |
| **Assessor(s)** | |  | | | | | | | |
| **Previous Assessment Date:** |  | | **This Assessment Date:** | **01/09/2023** | **Recommended Review Period:** | |  | | |

| **See Appendix for supplementary information on how to complete this risk assessment** | |
| --- | --- |
| **Description of Activity/Workplace/Equipment being assessed** | **Picture** |
| Brief description  This is an acute randomised, repeated measures cross-over design study. We will recruit 126 healthy adults living in East London from white, South Asian (SA) and black African-Caribbean (AC) origins.  Participants will consume a glucose drink (75g glucose in 300 ml water, 281 kcal) used for oral glucose tolerance test (OGTT) and pure orange juice (OJ, Tesco 100% Pure Squeezed Orange Juice Smooth 300 ml containing 129 kcal, 30 g sugar, 0.3 g fibre, 1.8 g protein and 90 mg vitamin C) on separate occasions with at least 48-hour interval and at random order. Participants come fasting at least eight hours (refer to Diabetes UK’s guideline). Blood glucose concentration is measured at 0, 30, 60, 90 and 120 min before and after drink consumption by finger prick by using HemoCue Glucose 201+ Analyser (HCU, Suffolk). A 7-ml fasting blood is collected via phlebotomy from the arm that will be centrifuged to separate plasma (Camlab CF0506 Clinical Centrifuge, Camlab Ltd, Cambridge. Picture 2). The plasma will be aliquoted to 1.5 ml Eppendorf tubes and stored at -20◦C freezer that is locked and only research staff can access. All Eppendorf tubes will be clearly labelled using freezer durable labels showing study date, participant ID and study visit.  After the study is completed, the plasma samples will be delivered to Coventry University to test biomarkers by University approved courier. The Safety Office will be contacted well in advance of transporting blood samples so that further guidance can be sought as necessary.  Drink preparation will take place at the staff kitchen using one-off cups and utensils on the morning of the experiment. The participants will consume the drinks at the foyer outside the lab. Participants will be called in to the lab only when blood samples are taken, and only one participant at a time.  The lab was used by Professor Danai Dima’s PhD students at the Department of Psychology in the past to take blood samples from participants via phlebotomy. The lab will be now exclusively used by our project and no other staff members apart from the research team members will access the lab during the study period until July 2024.  There will be a blood glucose analyser on the bench (Picture 1), an undercount fridge and an undercount freezer, a bench top centrifuge (Picture 2) in the lab. An office desk with a desk top computer and a chair are in the lab as well.  In addition, a sharps bin and biohazard bag will be in place to dispose the biohazardous waste. Used needles will be disposed in a sharp bin (Picture 3). The blood contaminated materials will be disposed at the clinical waste bag (Picture 4). The bench will be cleaned using CHLOR-CLEAN Chlorine Detergent after use.  The contract for disposal of sharps and all clinical waste at City St George’s, University of London is held by First Mile, specialist sub-contractor to Julius Rutherfoord who hold the contract for cleaning and waste disposal at City St George’s, University of London. Julius Rutherfoord is responsible for arranging collection and disposal of [clinical/hazardous/bacterial] waste in accordance with contract and agreed SLAs and completing and signing off on waste collection notes (SOP Clinical Waste was received and will be followed).  If the participants are faint due to blood phobia, or their fasting plasma glucose level is ≥ 11 mmol/L (participants’ fasting blood glucose level will be measured prior to taking test drinks), they will be asked to withdraw from the study for their safety purpose. If their fasting plasma glucose level is ≥ 7 mmol/L or their postprandial glycaemic response at 2 hour does not return to fasting level, they can remain in the study, but in all of the above situations, participants will be advised to contact their GP for further consultation. | **I** 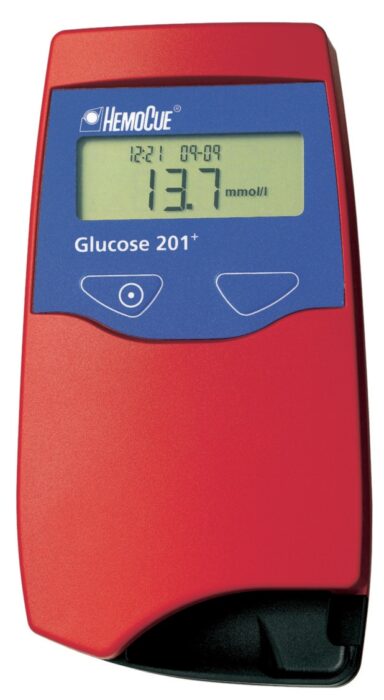  **Picture 1.** HemoCue Glucose 201+ Analyser  **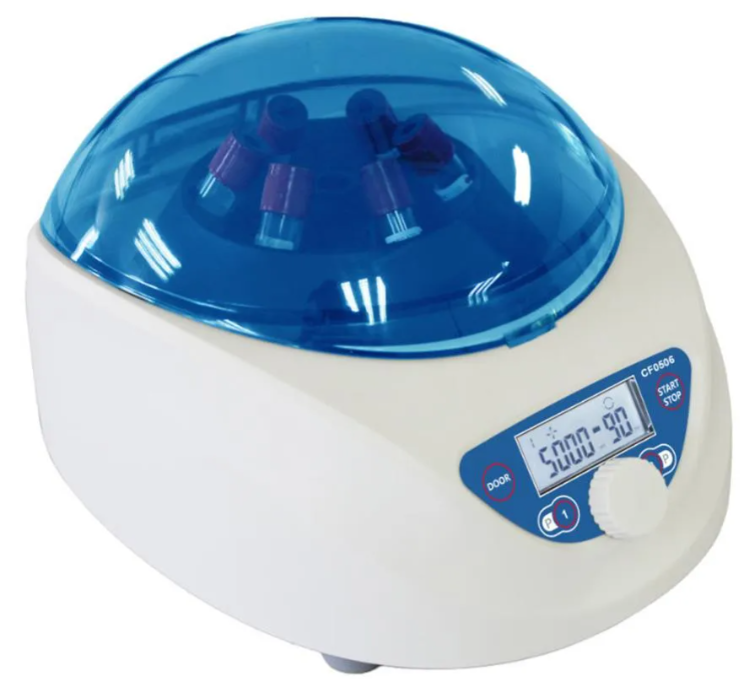**  **Picture 2. Camlab CF0506 Clinical Centrifuge with 6 x 15ml Rotor**  **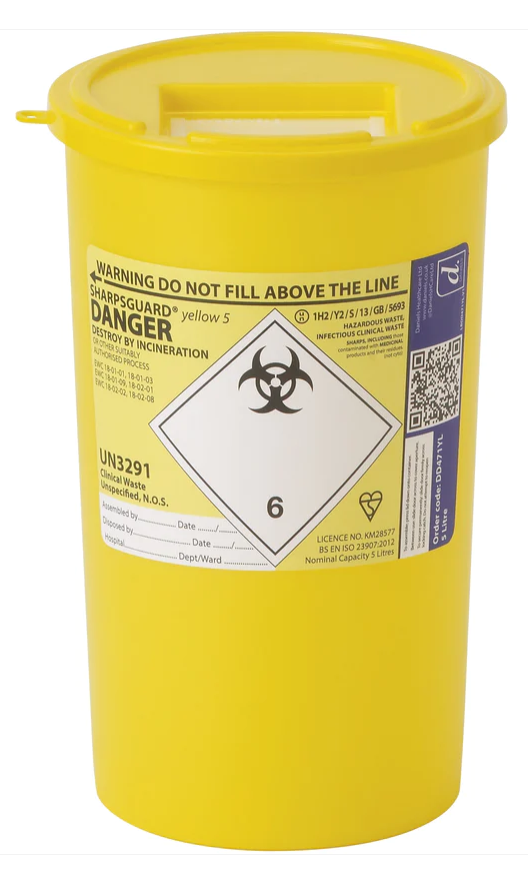 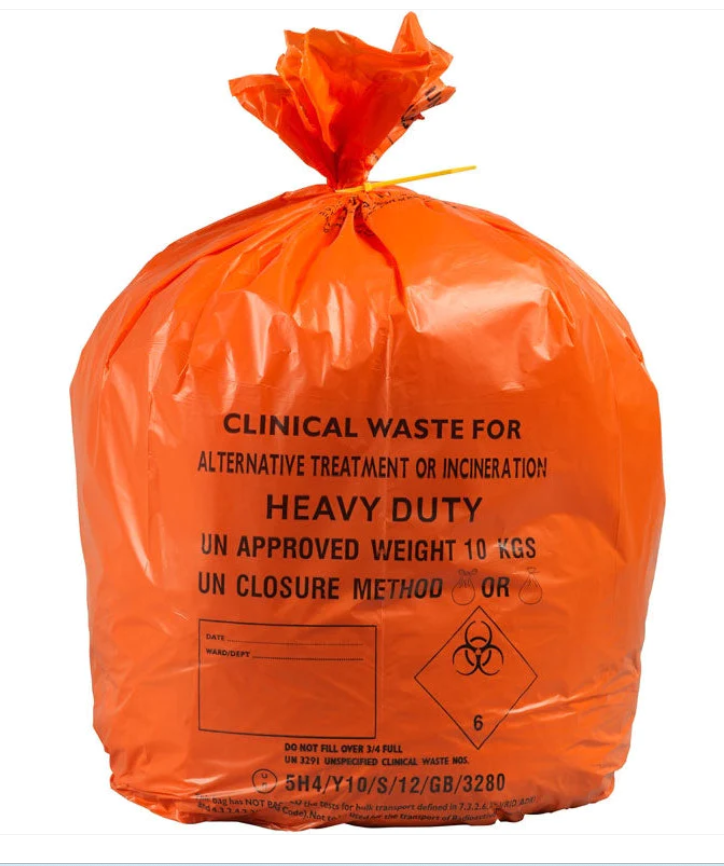**  **Picture 3. Sharp bin** Picture 4. Orange Medium Duty Clinical Waste Bags - 20L Small - Roll of 50 |

| **Hazard**  **No** | **HAZARD**  (potential for harm) Insert a description of the hazard and how harm might arise  e.g.  ***Use of Pillar Drill***   - *Electric Shock* - *Contact with moving parts* - *Impact from ejected objects* | **RISK^^[[1]](#footnote-1)^^**  **(without controls)** | | | **Groups of persons exposed to hazard** see appendix | **EXISTING CONTROL MEASURES**  (those already in place to reduce risk level)  e.g.   - *Electrical testing in date* - *Guarding in place, suitable and functional* | **RISK**  **(with**  **controls)** | | | ADDITIONAL CONTROLS REQUIRED?  (to reduce risk to acceptable level)  e.g.   - *All users require training in safe operation* |
| --- | --- | --- | --- | --- | --- | --- | --- | --- | --- | --- |
|  |  | Likelihood (L) | Severity (S) | Risk See Appendix |  |  | Likelihood (L) | Severity (S) | Risk see appendix |  |
|  | Bruise | **3** | **1** | **Low(3)** | **Participants** | Well trained researcher in phlebotomy  Press the needle area long enough with cotton pad | **3** | **1** | **Low(3)** | No |
|  | Fainting / Syncope episode (due to needle or blood phobia) | **2** | **1** | **Low(2)** | **Participants** | Participants who are fainting from needle or blood are not eligible for the study.  In case of a syncopal episode or a simple faint, we will follow the steps recommended by NHS about [Fainting](https://www.nhs.uk/conditions/fainting/#:~:text=If%20you're%20with%20someone,wake%20up%20within%2020%20seconds). | **1** | **2** | **Low(1)** |  |
|  | Blood borne disease, most notably Hepatitis B (HBV), Hepatitis C (HCV) and the Human Immunodeficiency Virus (HIV). | **2** | **5** | **Medium(10)** | **Researcher** | Well trained researcher in phlebotomy  Wear gloves, masks and lab goggles  Dealing with spillage of plasma/blood and cleaning bench after use:   - Wipe the area with water and detergent until it is visibly clean. - Saturate the area again with sodium hypochlorite 0.5% (10 000 ppm available chlorine). This is a 1:10 dilution of 5.25% sodium hypochlorite bleach, which should be prepared daily. - Rinse off the tongs, brush and pan, under running water and place to dry. - Remove gloves and discard them. - Wash hands carefully with soap and water, and dry thoroughly with single-use towels. | **1** | **5** | **Medium(5)** | Hepatitis B vaccination |
|  | Skin injury by sharps (e.g., needles) | **1** | **2** | **Low(2)** |  | According to [NHS guideline](https://www.nhs.uk/common-health-questions/accidents-first-aid-and-treatments/what-should-i-do-if-i-injure-myself-with-a-used-needle/), if you pierce or puncture your skin with a used needle, follow this first aid advice immediately:   - encourage the wound to bleed, ideally by holding it under running water - wash the wound using running water and plenty of soap - do not scrub the wound while you're washing it - do not suck the wound - dry the wound and cover it with a waterproof plaster or dressing - You should also seek urgent medical advice as you may need treatment to reduce the risk of getting an infection: - contact your employer's Occupational Health service if you injure yourself at work - otherwise call your GP, NHS 111 or go to the nearest [accident and emergency (A&E) department](https://www.nhs.uk/service-search/find-an-accident-and-emergency-service) | **1** | **2** | **Low(2)** |  |
|  | Blood/plasma spillage | **1** | **1** | **Low (1)** |  | We will follow the [Blood and Body Fluid Spillage Procedure](https://www.rdash.nhs.uk/wp-content/uploads/2017/08/2-Blood-and-Body-Fluid-Spillage-Procedure-v2.1.pdf) by Rotherham Doncaster and South Humber NHS Foundation Trust. The steps of dealing with blood spillage in this document is clear and easy to follow. | **1** | **1** | **Low (1)** |  |
|  |  |  |  |  |  |  |  |  |  |  |
|  |  |  |  |  |  |  |  |  |  |  |
|  |  |  |  |  |  |  |  |  |  |  |
|  |  |  |  |  |  |  |  |  |  |  |
|  |  |  |  |  |  |  |  |  |  |  |

| **Details of Personal Protective Equipment (P.P.E) identified as required for this activity** | | |
| --- | --- | --- |
| **Type** | **Specification** | **Available to all users and in good condition ✓/X** |
| **Lab gloves** | Stronghold Advanced Powder Free Nitrile Gloves | **✓** |
| **Eye Protection** | Chemical Safety Goggles | **✓** |
| **Face mask** | Disposable face masks | **✓** |

**Action Plan**

Ensure you include all additional controls required above.

| Hazard No. | Action required | Person Responsible and target date | Date Complete | Individual to sign off for completion of remedial action. Print name and sign |
| --- | --- | --- | --- | --- |
|  | No action needed |  |  |  |
|  | No action needed |  |  |  |
|  | Order required protection stuffs | Honglin Dong  1^st^ August 2023 | 10^th^ September 2023 | Honglin Dong |

| **Signature(s) of assessor(s)** | | **Signature of Head of Department/Principal Investigator responsible** | |
| --- | --- | --- | --- |
| Signed: |  | *I confirm that I have seen and approved this risk assessment form* | |
| Date: |  |  |  |
| Signed: |  | Name: | Honglin Dong |
| Date: |  | Signed: | Honglin Dong |
| Signed: | G Ward | Date: | 11/10/2023 |
| Date: | 11/10/2023x |  |  |

| **Name of SLO:** | **Gillian Ward** |
| --- | --- |

**Appendix:**

| **Guidance** | | |
| --- | --- | --- |
| - List any hazards that potentially exist - Use the risk matrix to assess the risk rating without any control measures in place. - Detail the control measures in place to control the hazard and reduce risk of injury or loss of another kind e.g. property damage - Use the matrix to assess the risk rating when the existing control measures are in place. - Complete an action plan for the additional control measures required to reduce risk to an acceptable level. - The highest risk rating is the highest single risk identified with existing control measures in place. | | |
| **Risk Matrix** | **Groups of Persons Exposed to Hazard** | |
| \|  \| \| **SEVERITY** \| \| \| \| \| \| --- \| --- \| --- \| --- \| --- \| --- \| --- \| \| **Slight**  **(No First aid no or little damage)** \| **Minor**  **(First aid injury minor damage )** \| **Moderate**  **(Medical treatment off site or property damage)** \| **Major**  **(Lost time accident/major injury or major damage)** \| **Very Severe**  **(Long term disability or fatality)** \| \| **1** \| **2** \| **3** \| **4** \| **5** \| \| **LIKELIHOOD** \| **Very Likely**  **(Common occurrence)**  **5** \| **Low (5)** \| **Medium(10)** \| **High (15)** \| **High (20)** \| **High (25)** \| \| **Likely**  **(Easily foreseeable)**  **4** \| **Low (4)** \| **Medium (8)** \| **Medium(12)** \| **High(16)** \| **High(20)** \| \| **Possible**  **(Foreseeable under unusual circumstances)**  **3** \| **Low(3)** \| **Low(6)** \| **Medium(9)** \| **Medium(12)** \| **High(15)** \| \| **Unlikely**  **(Unlikely sequence of events /**  **unplanned event)**  **2** \| **Low(2)** \| **Low(4)** \| **Low(6)** \| **Medium(8)** \| **Medium(10)** \| \| **Very Unlikely**  **1** \| **Low(1)** \| **Low(2)** \| **Low(3)** \| **Low(4)** \| **Low(5)** \| | Undergraduate  Postgraduate  Staff  Contractor  Visitor  Patient  General Public  Young Person  New/Expectant Mother  Disabled | Ug  Pg  S  C  V  Pa  Pu  Yp  Nm  D |

1. Please refer to the Risk Matrix in the Appendix to assign risk ratings [↑](#footnote-ref-1)
